# Shedding Light on Novel Pathogenic and Therapeutic Aspects Related to Immune-Mediated Skin Diseases

**DOI:** 10.3390/vaccines11040761

**Published:** 2023-03-29

**Authors:** Andrea Chiricozzi, Giampiero Girolomoni

**Affiliations:** 1 Dermatologia, Dipartimento di Medicina e Chirurgia Traslazionale, Università Cattolica del Sacro Cuore, 20123 Rome, Italy; 2 UOC di Dermatologia, Dipartimento di Scienze Mediche e Chirurgiche, Fondazione Policlinico Universitario A. Gemelli-IRCCS, 00168 Rome, Italy; 3 Section of Dermatology and Venereology, Department of Medicine, University of Verona, 37126 Verona, Italy

Great advances in the understanding of the pathogenic mechanisms characterizing various immune-mediated skin diseases have been achieved. As a consequence, new potential therapeutic targets have been identified. This Special Issue proposes insights on various immune-mediated disorders, shedding light on peculiar pathogenic features and novel therapeutic aspects that could be of great interest.

In common inflammatory skin disorders, including psoriasis and atopic dermatitis, whose pathogenic models have been mostly elucidated in the last two decades, the current research field is mostly oriented toward identifying novel treatment strategies or toward tailoring and personalizing treatment approaches. In this Special Issue, a pharmacogenomic approach to optimizing the use of biological agents, particularly ustekinumab, has been proposed, identifying 12 single-nucleotide polymorphisms (SNPs) in genes or allelic regions associated with an excellent response to ustekinumab [1]. Another chronic inflammatory skin disease, hidradenitis suppurativa (HS), is characterized by a complex and poorly defined pathogenesis that also includes alterations in the skin and, probably, gut bacterial composition. Due to the great diversity of methodologies that have been used to study the role of pathogens in HS, conflicting results have been collected so far. The state of the art has been explored by Rosi et al. [2]. DNA derived from bacteria, as well as from viruses, may promote inflammasome assembly and activation, with the secretion of IL-1β, initiating and sustaining the inflammatory response, potentially leading to autoinflammatory skin diseases such as HS. Non-self DNA is detected by certain cytoplasmatic pathogen sensors, such as AIM2, which is crucial in triggering IL-1β signals, involved in the pathogenesis of hidradenitis suppurativa, particularly in syndromic forms of hidradenitis suppurativa. A genetic variant of AIM2 was identified as a potential genetic susceptibility factor involved in the development of syndromic forms of HS but not involved in the development of sporadic HS and pyoderma gangrenosum [3].

The spectrum of immune-mediated skin diseases includes the immune-mediated skin manifestations induced by anti-SARS-CoV-2 vaccines. In this Special Issue, a case of Sweet syndrome, an acute febrile neutrophilic dermatosis, after the administration of the BNT162b2 vaccine is reported in a 52-year-old Caucasian male presenting with an acute febrile eruption that arose 72 h after his first dose of the BNT162b2 vaccine [4].

Overall, this Special Issue could represent a valuable tool in expanding the knowledge on the role of immunity in skin diseases in addition to the therapeutic relevance of targeting immune mediators.
